# Commissural alignment in transcatheter aortic valve replacement: A literature review

**DOI:** 10.3389/fcvm.2022.938653

**Published:** 2022-08-09

**Authors:** Abdul Muiz Khalid, Crochan J. O'Sullivan

**Affiliations:** College of Medicine and Health, University College Cork, Cork, Ireland

**Keywords:** TAVR, *commissures*, transcatheter and surgical aortic valve replacement, SAVR-surgical aortic valve replacement, alignment, THV, transcatheter heart valve

## Abstract

**Introduction:**

Transcatheter aortic valve replacement (TAVR) is a minimally invasive procedure to replace a diseased and faulty aortic valve in patients with severe aortic stenosis. As TAVR gains popularity among lower-risk younger patients with a longer life expectancy; there is a need to investigate the long-term shortcomings and limitations of the procedure for this patient group. One such shortcoming is that commissural alignment of transcatheter heart valves (THV) appears to be random; meaning that the THV neo-commissures can misalign with the native commissures of the aortic valve during deployment or self-expansion.

**Objectives:**

Identify techniques and procedures used to obtain commissural alignment in TAVR. Evaluate the effectiveness of these procedures in terms of the degree of commissural alignment. Analyse the impact of commissural alignment on coronary filling and re-access.

**Methods:**

Two electronic online databases were searched to identify existing literature relevant to the aim and objectives of this review: EBSCOhost and PubMed. After search filters were applied and duplicates removed; a total of 64 articles from both databases were screened against the inclusion/exclusion criteria. This resulted in a total of thirteen articles which met the objectives of this review and thus; were included.

**Results:**

All studies focused on a patient centered approach involving pre-TAVR computed tomography to obtain commissural alignment. Other studies modified this approach and combined techniques. All studies that implemented a technique to reduce commissural misalignment were significantly successful in obtaining commissural alignment when compared to a study in which alignment was random when no technique was implemented. Severe coronary overlapping in commissural aligned heart valves was relatively low compared to severe coronary overlapping when no technique was implemented.

**Conclusions:**

An increase in optimal commissural alignment *via* introduction of an alignment technique may seem attractive; however; the categorization of commissural alignment is arbitrary and does not accurately reflect real life clinical implications. Further research is needed to determine whether a routine procedure to achieve commissural alignment is necessary in low-risk younger patients undergoing TAVR.

## Introduction

Transcatheter aortic valve replacement (TAVR) is a minimally invasive procedure to replace a diseased and faulty aortic valve in patients with severe aortic stenosis (1). Previously; the standard for treatment of aortic stenosis was surgical (open heart) aortic valve replacement (SAVR); and unfortunately patients deemed as high-risk for surgery had limited options for treatment; such as diuretics; which only served as palliative care (2). TAVR was approved by the US Food and Drug Administration for high-risk surgical patients in 2012; allowing such patients an option for long term treatment. More recently; in 2019 it was approved for low-risk patients (2). In a randomized trial; TAVR was concluded to be superior to SAVR at 1 year in terms of deaths related to stroke or rehospitalization at an occurrence of 8.5% for TAVR and 15.1% for SAVR (3, 4). Other randomized trial studies either showed non-inferiority or superiority of TAVR over SAVR (4). The positive response from these trials combined with the fact that as many as 50% of patients with severe aortic stenosis are low risk for surgery has resulted in TAVR becoming the dominant treatment for aortic stenosis (3, 4). As TAVR becomes more popular for lower-risk younger patients with a longer life expectancy; there is a need to investigate the long-term shortcomings and limitations of the procedure for this patient group (3, 5).

One such shortcoming is that commissural alignment of transcatheter heart valves (THV) appears to be random; meaning that the THV neo-commissures can misalign with the native commissures of the aortic valve during deployment or self-expansion (5, 6). In contrast; bioprosthetic valves used in SAVR can be reliably aligned correctly; commissure-to-commissure with the native valve (7). In TAVR; the misalignment may result in the THV neo-commissures partially or fully overlapping the coronary artery ostia (5, 6). As a result; complications can arise if a younger patient needs a percutaneous coronary intervention or redo-TAVR especially since some of them will present with ischemic heart disease later in life (5, 7). With commissural misalignment there is also an increased risk of a leak during diastole because of the unnatural orientation of the THV; this may add undue stress to the THV leaflets (7). Improving commissural alignment in TAVR could possibly make subsequent coronary access and re-do TAVR easier; improve valve durability and enhance coronary blood flow (5, 7). The challenge lies in the fact that aortic anatomy as well as native orientation of the aortic valve differs in every patient; thus a universal approach would not suffice (5). Little attention has been paid to commissural alignment in clinical practice and no official instructions from THV manufacturers exist to achieve commissural alignment (5). Major randomized trials comparing SAVR and TAVR have not considered this limitation either (5).

### Aim

The aim of this study is to review published literature on procedures used to obtain commissural alignment in TAVR.

### Objectives

1. Identify techniques and procedures used to obtain commissural alignment in TAVR.
2. Evaluate the effectiveness of these procedures in terms of the degree of commissural alignment.
3. Analyse the impact of commissural alignment on coronary filling and re-access.

## Methods

**Figure 1:**
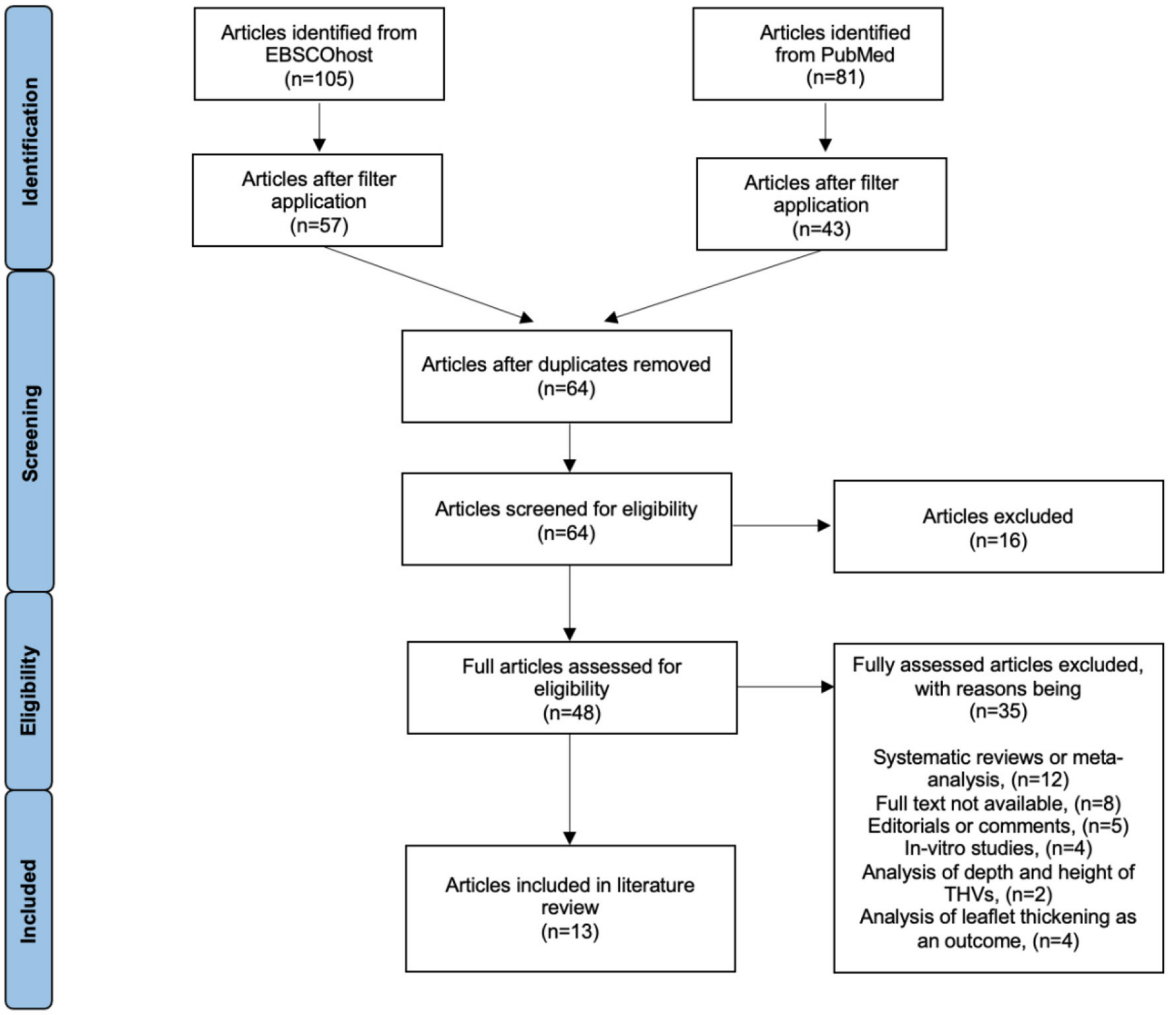
PRISMA flow diagram for selection process of relevant articles.

Two online databases were searched; including PubMed and EBSCOhost (all databases within EBSCOhost were selected); with a publication date range of 2014 to February 2022. The search terms “Transcatheter Aortic Valve Replacement” [Mesh] and “commissural alignment” were utilized in the search strategy. Search filters were applied to reflect the following: articles published in English available in full text with the subjects as adult humans. One reviewer independently identified articles investigating techniques to achieve commissural alignment in TAVR and screened them against the inclusion/exclusion criteria (see appendix A). A summary of the article selection process is given in Figure 1 according to the PRISMA statement. Reference lists of articles were searched to identify any missed studies and relevant articles were included. The quality of individual studies was assessed using the EBL critical appraisal checklist for quantitative studies and the CASP critical appraisal tool for qualitative studies (see appendix B and C). Each study was approved by its local medical ethics committee.

## Results

The data extracted from the articles reviewed are detailed in the summary Table 1 below.

**Table 1:** Summary of articles reviewed.

**Author(s); (Year); Location; Title**	**Study population; Sample size; Selection criteria**	**Study type; Design**	**Key findings**
Bieliauskas et al. (2021), Denmark Patient-Specific Implantation Technique to Obtain Neo-Commissural Alignment with Self-Expanding Transcatheter Aortic Valves (5).	*n* = 60 -Symptomatic SAS -Mean age 79 Exclusion: Non-transfemoral access; bicuspid aortic valve or renal impairment; balloon expandable THVs.	Non-randomized control trial (quasi-experiment) -3 different THV platforms (Evolut R/PRO; ACURATE neo2 and Portico-−20 patients in each group). -Patients underwent CT twice; 3 months before and after TAVR. -Preprocedural CT was used to determine fluoroscopic projection of RCC/LCC cusp overlap view. - Patient-specific implantation technique was based on THV fluoroscopic ‘marker’ and patient's CT. - Postprocedural CT used to assess commissural alignment (aligned; mild; moderate; severe).	- Mild CMA (<30°) obtained in 53 patients (88%); of which optimal commissural alignment (<15°) was obtained in 36 patients (60%). - Severe CMA (>45°) obtained in 2 patients. -In patients where the fluoroscopic projection was optimally assessed; the success rate of TAVR with optimal alignment or mild CMA was 98%. - The ACURATE neo2 platform produced mild CMA in all 20 cases. - Mild valvular leak was detected in 22 patients.
Fuchs et al. (8); Denmark and USA Commissural Alignment of Bioprosthetic Aortic Valve and Native Aortic Valve Following Surgical and Transcatheter Aortic Valve Replacement and its Impact on Valvular Function and Coronary Filling (8).	*n* = 240 -Symptomatic SAS -Mean age 80 Exclusion: Bicuspid aortic valve or renal impairment.	Non-randomized control trial (quasi-experiment) -28 patients underwent SAVR; and 212 underwent TAVR. - Commissural orientation was assessed pre- and post-AVR within 3 months; CT scans were analyzed separately. - Commissural alignment was calculated using three angles; measured using CT images; into one mean angle deviation. - Commissural alignment was defined as aligned (angle deviation of <15°); mild CMA (15° to 30°) moderate CMA (30° to 45°) and severe CMA (>45°).	−27 of 28 (96%) SHVs were implanted as aligned. One SHV was implanted with mild CMA. −47 THVs (22%) were implanted as aligned; 53 THVs (25%) were implanted with mild CMA; 46 (22%) had moderate CMA and 66 (31%) had severe CMA. - Commissural alignment in TAVR was shown to be random. - Severe CMA did not result in significant pressure drop within coronary arteries when compared to aligned THVs (0.9 RCA; 1.7 LCA)
Tang et al. (6); Denmark and USA Alignment of Transcatheter Aortic-Valve Neo-Commissures (ALIGN TAVR) (6).	*n* = 828 (483 SAPIEN 3; 245 Evolut; and 100 ACURATE-neo valves) -Symptomatic SAS -Mean age 80.2	Non-randomized control trial (quasi-experiment) -Pre-TAVR CT imaging was overlapped with fluoroscopic views using 3mensio software to map out the neo-commissural alignment of THVs with native commissures. -Coronary artery overlap was characterized as severe if a THV commissure and coronary ostium were 0° to 20° apart.	-Tracking Evolution Hat marker reduced the coronary artery overlap by 36% to 60% (*p* < 0.05). - More than 30% to 50% of cases had CMA where overlap with one or both coronary arteries were present.
Redondo et al. (7); Spain Accurate Commissural Alignment during ACURATE neo–TAVI Procedure (7).	*n* = 11 -Symptomatic SAS Inclusion: Patients treated with ACURATE-neo THV.	Non-randomized control trial (quasi-experiment) - Computer simulated “in silico” model was developed to predict final THV commissure orientations based on analysis of pre-TAVR CT images. -Patients underwent TAVR guided by computer simulated model. Commissural alignment analyzed using post-TAVR CT.	- None of the 9 accurate commissural alignment cases reported significant coronary artery overlap (0° to 20° apart was deemed sever overlap) - 7 out of 11 cases were aligned (angle deviation of <15°); 1 case had mild CMA (15° to 30°); and the remaining 2 cases had moderate CMA (30° to 45°); as predicted in computer simulation.
De Marco et al. (9); Italy A Patient-Specific Algorithm to Achieve Commissural Alignment with ACURATE-Neo: The Sextant Technique (9).	n = 45 -Symptomatic SAS -Mean age 81.6 Inclusion: Transfemoral TAVR using the ACURATE-neo2 THV.	Non-randomized control trial (quasi-experiment) -Pre- and post-TAVR CT used to calculate an internal bisector of the angle between coronary arteries +/- 15° error range. -THV was rotationally deployed so that one of the THV commissures aligns with the internal bisector. -Commissural alignment was defined as aligned (angle deviation of <15°); mild CMA (15° to 30°) moderate CMA (30° to 45°) and severe CMA (>45°).	-Coronary clearance was achieved in 98% of patients. -No cases of severe coronary artery overlap. - Commissural alignment was achieved in 34 (75.5%) patients; mild CMA in 9 (20%) patients and moderate CMA in 2 (4.5%) patients. - In 42 (93%) patients; final alignment of THV commissures were consistent with the calculated alignment with a mean difference of 10.5°± 5.2°.
Ochiai et al. (10); USA Coronary Access After TAVR (10).	n = 428 -Symptomatic SAS −66 treated with Evolut R/PRO −345 treated with SAPIEN 3 Exclusion: Poor quality CT images.	Non-randomized control trial (quasi-experiment) -Distance from inflow of the THV to the coronary ostium was measured. -Overlap between THV commissures and the coronary arteries was assessed using post-TAVR CT. -Coronary access deemed as unfavorable if the coronary ostium was in front of the THV commissures above or below the skirt in either artery.	- Unfavorable coronary access was observed in 34.8% for the left coronary artery and 25.8% for the right coronary artery in the Evolut PRO/R group (40 total) -Unfavorable coronary access was observed in 15.7% for the left coronary artery and 8.1% for the right coronary artery in the SAPIEN 3 group (82 in total). -The success rates for CAG or PCI post-TAVR were significantly lower in patients deemed as unfavorable for coronary access in both groups.
Holzamer et al. (11); Germany Multislice Computed Tomography-based Prediction of The	*n* = 244 -Symptomatic SAS Exclusion: Bicuspid aortic valves and valve-in-valve TAVR.	Non-randomized control trial (quasi-experiment) -The line of perpendicularity and implanter views were calculated using multiple plane reconstructions of the patients' pre-TAVR multislice CT scans.	- In 237 patients (97%); the angiogram was able to confirm the predicted line of perpendicularity and predict the optimal orientation of the THV.
Implantation Plane In Transcatheter Aortic Valve Implantation: Determination of The Line Of Perpendicularity and the Implanter's Views (11).	Bicuspid aortic valve; TAV-in-TAV; or poor quality CTs.	-The line of perpendicularity was confirmed by aortic angiogram and was used to orientate the THV allowing for corrections in commissural alignment.	−7 patients (3%) needed subsequent corrections to achieve optimal alignment; largest correction was 14°.
Abdelghani M, et al. (12); Germany Coronary Access After TAVR With a Self-Expanding Bioprosthesis: Insights from Computed Tomography (12).	*n* = 101 -Symptomatic SAS -Mean age 81.5 -Evolut PRO/R Exclusion:	Non-randomized control trial (quasi-experiment) - Post-TAVR multislice CT scans (30 days after) were used to assess interference of THV commissures with coronary access. -Included measuring the longitudinal distance from the THV out flow to inflow to the center of the RCA and LCA ostia and the transverse distance between the THV commissures and the coronary ostium.	- The THV commissures vertically aligned with the coronary ostium were 58% and 63% in the RCA and LCA; respectively. - THV commissures were not aligned with the native commissures in 45 patients (47%) - The commissural posts were overlapping a coronary ostium in 15 patients (16%). - Two patients (2%) had a paravalvular leak due to CMA; caused by the sealing skirt.
Rogers et al. (13); USA Feasibility of Coronary Access and Aortic Valve Reintervention in Low-Risk TAVR Patients (13).	*n* = 137 -Symptomatic SAS -Mean age 73.8 Inclusion: Patients treated with the SAPIEN 3 THV.	Non-randomized control trial (quasi-experiment) - Post-TAVR CT scan (30 days after) scans were used to observe the transverse distance between the THV commissures and the coronary ostium. - In a subgroup; intentional crimping of the THV catheter was tested to pre-determine commissural alignment.	- THV commissures overlapping a coronary ostium was observed in 9% to 13% of patients. - Intentional crimping did not significantly impact commissural alignment; orientation of the commissures away from the coronary ostium was achieved in 75% (15/20 patients) who were treated with intentionally crimped alignment compared to 70.3% (45/64 patients) treated with randomly crimped catheters (p=0.69).
Buono et al. (14); Italy Commissural Alignment with New-generation Self-expanding Transcatheter Heart Valves During Aortic Replacement (14).	*n* = 4 - Symptomatic SAS -Mean age 80.75 -One patient treated with	Retrospective case report series - Pre-TAVR multislice CT angiography used to construct angiographic projections in cusp overlap and coplanar views. - Cardiac and vascular structures were analyzed using 3mensio software.	- Commissural alignment was achieved in all circumstances as coronary artery re-cannulation was easily obtained in all cases.
	ACURATE-neo 2; Evolut R; Portico and NVT Allegra.		
Redondo et al. (15); Spain Commissural vs. Coronary Optimized Alignment During Transcatheter Aortic Valve Replacement (15)	*n* = 100 -Symptomatic SAS Exclusion: Bicuspid aortic valve and poor quality imaging.	Observational (cross-sectional) study -Pre-TAVR CT used to measure distance from native commissures to the RCA and LCA determining eccentricity. -THV virtually placed using simulation with ideal commissural alignment and the degree of coronary overlap was classified. - Three groups defined for coronary overlap: no risk (>35° from neocommissures to coronary ostia); moderate risk (20°-35°); and severe risk (≥20°).	−32 patients had moderate to severe risk of coronary overlap regardless of ideal commissural alignment. - Greater coronary eccentricity was linked with greater risk of moderate to severe coronary overlap regardless of commissural alignment. - When optimal coronary alignment was simulated; it reduced severe coronary overlap in all cases and reduced moderate coronary overlap by 22%.
Tang et al. (16); USA Conventional vs. Modified Delivery System Technique in Commissural Alignment from the Evolut low-risk CT Substudy (16).	*n* = 249 conventional technique patients n =240 modified technique patients - Symptomatic SAS Inclusion: Patients in the Evolut Low Risk LTI substudy.	Non-randomized control trial (quasi-experiment) - Patients underwent high-quality electrocardiographically synchronized CT scans. - Conventional technique patients had the delivery system inserted into femoral artery with flush port at 12 o'clock orientation. Modified technique patients had a 3 o'clock orientation. - Severe coronary artery overlap was defined as 0° to 20°.	- The modified technique had improved commissural alignment and reduced severe coronary artery overlap. - Outer curve hatmaker rate was 89.6% for the modified technique and 67.5% for the conventional. - Out of the conventional technique cohort; 41.6% had severe coronary overlap with the Evolut valve commissure in 1 or both coronary arteries; compared to a reduced 20.8% coronary overlap in 1 or both coronaries using the modified technique.
Tarantini et al. (17); Italy Coronary Access After Transcatheter Aortic Valve Replacement with Commissural Alignment: The ALIGN-ACCESS Study (17).	*n* = 206 - Symptomatic SAS Inclusion: Patients treated with SAPIEN 3; Evolut R/Pro; or Acurate Neo THVs.	Non-randomized control trial (quasi-experiment) - Coronary angiography was performed after TAVR. - 38% of patients received SAPIEN 3; 31.1% Evolut Pro/R and 30.1% Acurate Neo THVs. - Evolut THVs were implanted with an aim of commissural alignment and Acurate Neo THVs were retrospectively assessed to achieve commissural alignment.	- Commissural alignment was achieved in 85.9% of Evolut and 69.4% of Acurate Neo cases. - Coronary access was higher in SAPIEN 3 than both Evolut and Neo THVs regardless of whether they were aligned or misaligned (95% vs. 71% and 46%; respectively). - Cannulation of at least 1 coronary artery was unfeasible in 0% for SAPIEN; 11% in misaligned supr-annular and 3% aligned supra-annular THVs.

### Objective 1: Techniques and procedures used to obtain commissural alignment in TAVR

All studies focused on a patient centered approach to obtain commissural alignment. The most common technique to obtain commissural alignment was a patient-centered approach involving pre-TAVR multidetector or multislice CT to create or calculate a projection of the THV on the native aortic valve which may predict the final valve orientation. This was the core methodology used in nine studies (5–11, 14, 15). A subgroup of these studies modified this technique further and combined the CT imaging with fluoroscopic imaging to identify “markers” on the frame of the THV platforms which could be used to align THV and native commissures (5–8). A few studies modified the approach and used computer simulations using a specialized software (3mensio) to create projections onto CT images in order to minimize coronary artery overlap by predicting the optimal orientation for commissural alignment (6, 7, 14, 15). Another technique used was crimping of the THV on to the delivery catheter suited to the aortic anatomy of the patients based on pre-calculated commissural alignment (6, 13).

### Objective 2: The effectiveness of these procedures in terms of the degree of commissural alignment

Many studies used a consistent method of assessing the extent of commissural misalignment (CMA) which involved categorizing the alignment into angle deviations (5–9). The categories were: aligned (angle deviation of <15°); mild CMA (15° to 30°) moderate CMA (30° to 45°) and severe CMA (>45°). Within the low to high quality studies that assessed this aspect after implementation of alignment techniques; very few patients had severe CMA after TAVR; ranging from 0% to 3.3% of their respective populations (5, 7, 9). However; the occurrence of mild and moderate CMA was more variable between the studies (5, 7, 9). The occurrence of correct alignment was high and occurred in more than half the population in all three studies assessing this aspect; ranging from 60% to 75.5% of their respective populations (5, 7, 9) Comparatively; the study by Fuchs et al. in which techniques to achieve commissural alignment were not implemented; the distributions of occurrences of the alignment categories were found to be random in TAVR (8).

### Objective 3: Impact of commissural alignment on coronary filling and re-access

Five studies addressed the impact of commissural alignment on coronary blood flow and subsequent intervention in the form of coronary angiography or PCI. In these studies; severe overlap of either the RCA or LCA with the THV commissures was classified if a THV commissure and coronary ostium were 0° to 20° apart (6, 8, 10, 12, 13). The results were variable as severe overlapping occurred in a range of 9% to 16% of the patients in their respective populations within the various studies (8, 10, 12–14). This was still relatively low compared to severe coronary overlapping when no technique to align commissures was implemented; as in the case of the study by Fuchs et al. in which 50% of cases had severe coronary overlapping. One study found that severe CMA did not result in significant pressure drop within coronary arteries when compared to aligned THVs (8). The success rates for CAG or PCI post-TAVR were significantly lower in patients deemed as unfavorable for coronary access (10). When commissural alignment was achieved successfully; coronary intervention was possible in all cases (14).

## Discussion

All studies that implemented a technique to reduce commissural misalignment were significantly successful in obtaining commissural alignment when compared to the study by Fuchs et al. in which alignment was random when no technique was implemented. An increase in optimal commissural alignment of 22% (8) to 75.5% (10) via introduction of an alignment technique may seem attractive; however; one major shortcoming of these results was that classification of commissural alignment was artificial and did not reflect the clinical implications of even mild CMA. There was no method to measure differences in clinical implications of CMA in slight adjustments of THV orientation by 1° angle deviation in either direction. Thus; the categorization of commissural alignment was arbitrary and does not accurately reflect real life clinical implications.

Another major shortcoming of the results was that an angle deviation of <15° was considered as commissural alignment; whereas severe coronary artery overlap was defined if a THV commissure and coronary ostium were 0° to 20° apart. This means that if the THV was aligned with the native commissures of the aortic valve at 15°; a slight overlap of even one of the commissures with either coronary artery could be deemed as severe coronary overlap and thus coronary intervention unfeasible. These contradictory classifications between studies open the question for validity and the clinical implications of these studies. Post-TAVR intervention feasibility was hypothetical in most studies and was only carried out in a clinical scenario within a subset of the sample population treated. Thus; the validity and application to real world scenarios on a larger scale is questionable.

Comparisons between commissural alignment achieved in the different THV platforms were drawn in a few studies and the results from Ochiai et al. (10) and Tarantini et al. (17) may indicate that the SAPIEN 3 platform is favorable to commissural alignment and subsequent coronary access as patients receiving this platform had considerably lower percentages of unfavorable coronary access. For example; when compared to the Evolut and Acurate Neo platforms; coronary access was higher in SAPIEN 3 than both Evolut and Neo THVs regardless of whether they were aligned or misaligned (95% vs. 71% and 46%; respectively). This is however contradictory to the findings of the study by Bieliauskas et al. (5) in which the Acurate Neo platform had the highest rate of achieving mild CMA compared to others. It is too early to determine which platform is best suited for commissural alignment as they require different techniques to achieve commissural alignment and each patient is individual in the selection of the THV platform based on their clinical situation; comorbidities; anatomy of the aortic root and the potential access route (18).

Due to the recency of commissural alignment in TAVR; a clear gap can be identified in the research area; which is that the long-term implications of commissural alignment have not been studied. From the lack of long-term studies; cardiologists around the world are not sure if commissural alignment is if even worth adopting as a routine procedure for low-risk younger patients with aortic stenosis as the long-term benefits or complications are not known. A cohort study design where low risk patients who have undergone TAVR using a commissural alignment technique are observed for a minimum of 10 years is needed to justify the adoption of commissural alignment as a routine procedure.

The limitations of this review include the relative recency of commissural alignment in TAVR as a topic; thus the articles reviewed had very similar study designs and lacked control groups to compare outcomes such as coronary filling and intervention. This also means that the data extracted from the articles was very similar and could easily be interpreted as a continuous study. On the other hand; the relative recency of this topic also creates the strengths of this literature view as since the studies available are limited; a comprehensive review was able to be carried out covering most of the literature available for the topic at hand.

## Author contributions

AK is the primary author and CO'S is the project supervisor. Both authors listed have made a substantial, direct, and intellectual contribution to the work and approved it for publication.

## Conflict of interest

The authors declare that the research was conducted in the absence of any commercial or financial relationships that could be construed as a potential conflict of interest.

## Publisher's note

All claims expressed in this article are solely those of the authors and do not necessarily represent those of their affiliated organizations; or those of the publisher; the editors and the reviewers. Any product that may be evaluated in this article; or claim that may be made by its manufacturer; is not guaranteed or endorsed by the publisher.

## Abbreviations

TAVR: Transcatheter Aortic Valve Replacement
SAVR: Surgical Aortic Valve Replacement
SAS: Severe Aortic Stenosis
THV: Transcatheter Heart Valve
SHV: Surgical Heart Valve
LCC/RCC: Left Coronary Cusp/Right Coronary Cusp
CT: Computed Tomography
CMA: Commissural Misalignment
RCA/LCA: Right Coronary Artery/Left Coronary Artery
Percutaneous Coronary Intervention: PCI
CAG: Coronary Angiography.

## References

[1] Popma JJ Deeb GM Yakubov SJ Mumtaz M Gada H O'Hair D. Transcatheter aortic-valve replacement with a self-expanding valve in low-risk patients. N Engl J Med. (2019) 380:1706–15. 10.1056/NEJMoa1816885 30883053

[2] Mahmaljy H Tawney A Young M. Transcatheter aortic valve replacement. In: StatPearls. Treasure Island (FL): StatPearls Publishing (2021). 28613729

[3] Hanzel GS Gersh BJ. Transcatheter aortic valve replacement in low-risk, young patients. Circulation. (2020) 142:1317–9. 10.1161/CIRCULATIONAHA.120.047874 33017211

[4] Kumbhani D. TAVR is not the ‘beginning of the end’ for Aortic Stenosis Open Heart Surgery. Heart UT Southwestern Medical Center. Available online at: http://utswmed.org/medblog/tavr-not-beginning-end-aortic-stenosis-open-heart-surgery/ (accessed November 13, 2021).

[5] Bieliauskas G Wong I Bajoras V Wang X Kofoed KF De Backer O. Patient-Specific implantation technique to obtain neo-commissural alignment with self-expanding transcatheter aortic valves. JACC Cardiovasc Interv. (2021) 14:2097–108. 10.1016/j.jcin.2021.06.033 34538602

[6] Tang GHL Zaid S Fuchs A Yamabe T Yazdchi F Gupta E. Alignment of Transcatheter Aortic-Valve Neo-Commissures (ALIGN TAVR): impact on final valve orientation and coronary artery overlap. JACC Cardiovasc Interv. (2020) 13:1030–42. 10.1016/j.jcin.2020.02.005 32192985

[7] Redondo A Valencia-Serrano F Santos-Martínez S Delgado-Arana JR Barrero A Serrador A. Accurate commissural alignment during ACURATE neo TAVI procedure. Proof of concept. Rev Esp Cardiol (Engl Ed). (2021) 75:203–12. 10.1016/j.rec.2021.02.004 33781722

[8] Fuchs A Kofoed KF Yoon S-H Schaffner Y Bieliauskas G Thyregod HG. Commissural alignment of bioprosthetic aortic valve and native aortic valve following surgical and transcatheter aortic valve replacement and its impact on valvular function and coronary filling. JACC Cardiovasc Interv. (2018) 11:1733–43. 10.1016/j.jcin.2018.05.043 30121280

[9] De Marco F Casenghi M Spagnolo P Popolo Rubbio A Brambilla N Testa L. A patient-specific algorithm to achieve commissural alignment with Acurate Neo: the sextant technique. Catheter Cardiovasc Interv. (2021) 98:E847–54. 10.1002/ccd.29737 33960624 PMC9292557

[10] Ochiai T Chakravarty T Yoon S-H Kaewkes D Flint N Patel V. Coronary access after TAVR. JACC Cardiovasc Interv. (2020) 13:693–705. 10.1016/j.jcin.2020.01.216 32192689

[11] Holzamer A Sitka E Hengstenberg C Schmid C Debl K Maier L. Multislice computed tomography-based prediction of the implantation plane in transcatheter aortic valve implantation: determination of the line of perpendicularity and the implanter's views. Eur J Cardiothorac Surg. (2015) 48:879–85. 10.1093/ejcts/ezv095 25825262

[12] Abdelghani M Landt M Traboulsi H Becker B Richardt G. Coronary access after TAVR with a self-expanding bioprosthesis: insights from computed tomography. JACC Cardiovasc Interv. (2020) 13:709–22. 10.1016/j.jcin.2020.01.229 32192691

[13] Rogers T Greenspun BC Weissman G Torguson R Craig P Shults C. Feasibility of coronary access and aortic valve reintervention in low-risk TAVR patients. JACC Cardiovasc Interv. (2020) 13:726–35. 10.1016/j.jcin.2020.01.202 32192693

[14] Buono A Morello A Pero G Corcione N Bettari L Saccocci M. Commissural alignment with new-generation self-expanding transcatheter heart valves during aortic replacement. Cardiovasc Revasc Med. (2021). 10.1016/j.carrev.2021.07.027. [Epub ahead of print]. 34362686

[15] Redondo A Baladr ón ZC Tch étché D Santos -Martinez Sandra Delgado -Arana Jose Raúl Barrero A. Commissural versus coronary optimized alignment during transcatheter aortic valve replacement *JACC Cardiovas Interv*. (2022) 15:135–46. 10.1016/j.jcin.2021.10.005 35057983

[16] Tang GHL Sengupta A Alexis SL Zaid S Leipsic JA Blanke P. Conventional versus modified delivery system technique in commissural alignment from the evolut low-risk CT substudy. Catheter Cardiovasc Interv. (2022) 99:924–31. 10.1002/ccd.29973 34626449

[17] Tarantini G Nai Fovino L Scotti A Massussi M Cardaioli F Rodinò G. Coronary access after transcatheter aortic valve replacement with commissural alignment: the ALIGN-ACCESS study. Circ Cardiovasc Interv. (2022) 15:e011045. 10.1161/CIRCINTERVENTIONS.121.011045 35167332

[18] Renker M Kim WK. Choice of transcatheter heart valve: should we select the device according to each patient's characteristics or should it be “one valve fits all”? Ann Transl Med. (2020) 8:961. 10.21037/atm.2020.04.13 32953761 PMC7475391

