# Supplementary material for: Commissural alignment in transcatheter aortic valve replacement: A literature review
Source: Front Cardiovasc Med. 2022 Aug 9;9:938653. doi: 10.3389/fcvm.2022.938653 (PMC9396239; doi:10.3389/fcvm.2022.938653)
Supplement: Supplementary file 1 [file Data_Sheet_1.pdf]

Appendices

Appendix A: Inclusion and Exclusion Criteria for Articles included in the Literature Review

Inclusion Criteria	Exclusion Criteria
Studies published between 2014 and February 2022.	Studies not published in peer reviewed academic journals.
Original research studies that are quantitative or qualitative including: randomised controlled trials, case-control, cohort, cross-sectional and case reports.	Studies that are systematic reviews, literature reviews, editorials, meta-analyses, commentaries, letters, or imaging vignettes.
Studies on adults (aged 19 and over).	Studies on pediatric patients.
Studies on humans.	Studies on species other than humans or done in-vitro.
Studies on patients with severe aortic stenosis undergoing TAVR.	Studies on patients with aortic regurgitation.
Studies that compare commissural alignment in TAVR to alignment in SAVR in terms of coronary flow and/or re-access.	Studies focused solely on commissural alignment in SAVR.
Studies that assess coronary blood flow, redo-TAVR, valvular function or percutaneous coronary intervention as outcomes.	Studies that assess leaflet thickening or leaflet laceration as outcomes.
Studies that classify the degree of commissural misalignment as severe, mild, or low with quantifiable characteristics.	Studies that analyse depth, height, or anything other than orientation of THVs in TAVR.

Appendix B: Details of EBL Critical Appraisal Checklist for Quantitative Studies Reviewed

EBL Critical Appraisal Checklist		Bieliauskas et al.	Fuchs et al.	Tang et al.	Redondo et al.	De Marco et al.	Ochiai et al.	Holzamer et al.	Abdelghani et al.	Rogers et al.	Redondo et al.	Tang et al.	Tarantini et al.
**Section A: Population**	Is the study population representative of all users, actual and eligible, who might be included in the study?	Y	Y	Y	Y	Y	Y	Y	Y	Y	Y	Y	Y
	Are inclusion and exclusion criteria definitively outlined?	Y	Y	N	N	N	Y	Y	Y	N	Y	Y	Y
	Is the sample size large enough for sufficiently precise estimates?	N	N	Y	N	N	Y	N	N	N	N	Y	N
	Is the response rate large enough for sufficiently precise estimates?	N/A	N/A	N/A	N/A	N/A	N/A	N/A	N/A	N/A	N/A	N/A	N/A
	Is the choice of population bias-free?	N	N	N	N	N	N	N	N	N	N	N	N
	If a comparative study: Were participants randomized into groups? Were the groups comparable at baseline? If groups were not comparable at baseline, was incomparability addressed by the authors in the analysis?	N/A	N/A	N/A	N/A	N/A	N/A	N/A	N/A	N/A	N/A	N/A	N/A
		Y	Y	Y	Y	Y	Y	Y	Y	Y	Y	Y	Y
		N/A	N/A	N/A	N/A	N/A	N/A	N/A	N/A	N/A	N/A	N/A	N/A
		N/A	N/A	N/A	N/A	N/A	N/A	N/A	N/A	N/A	N/A	N/A	N/A
	Was informed consent obtained?	Y	Y	Y	Y	Y	Y	Y	Y	Y	Y	Y	Y
**Section B: Data Collection**	Are data collection methods clearly described?	Y	Y	Y	Y	Y	Y	Y	Y	Y	Y	Y	Y
	If a face-to-face survey, were inter-observer and intra-observer bias reduced?	N/A	N/A	N/A	N/A	N/A	N/A	N/A	N/A	N/A	N/A	N/A	N/A
	Is the data collection instrument validated?	Y	Y	Y	Y	Y	Y	Y	Y	Y	Y	Y	Y
	If based on regularly collected statistics, are the statistics free from subjectivity?	N/A	N/A	N/A	N/A	N/A	N/A	N/A	N/A	N/A	N/A	N/A	N/A
	Does the study measure the outcome at a time appropriate for capturing the intervention's effect?	Y	Y	Y	Y	Y	Y	Y	Y	Y	Y	Y	Y
	Is the instrument included in the publication?	Y	Y	Y	Y	Y	Y	Y	Y	Y	Y	Y	Y
	Are questions posed clearly enough to be able to elicit precise answers?	Y	Y	Y	Y	Y	Y	Y	Y	Y	Y	Y	Y
	Were those involved in data collection not involved in delivering a service to the target population?	Y	Y	Y	Y	Y	Y	Y	Y	Y	Y	Y	Y
**Section C: Study Design**	Is the study type / methodology utilized appropriate?	Y	Y	Y	Y	Y	Y	Y	Y	Y	Y	Y	Y
	Is there face validity?	Y	Y	Y	Y	Y	Y	Y	Y	Y	Y	Y	Y
	Is the research methodology clearly stated at a level of detail that would allow its replication?	Y	Y	Y	Y	Y	Y	Y	Y	Y	Y	Y	Y
	Was ethics approval obtained?	Y	Y	Y	Y	Y	Y	Y	Y	Y	Y	Y	Y
	Are the outcomes clearly stated and discussed in relation to the data collection?	Y	Y	Y	Y	Y	Y	Y	Y	Y	Y	Y	Y
**Section D: Results**	Are all the results clearly outlined?	Y	Y	Y	Y	Y	Y	Y	Y	Y	Y	Y	Y
	Are confounding variables accounted for?	Y	Y	Y	Y	Y	N	Y	Y	Y	Y	Y	Y
	Do the conclusions accurately reflect the analysis?	Y	Y	Y	Y	Y	Y	Y	Y	Y	Y	Y	Y
	Is subset analysis a minor, rather than a major, focus of the article?	Y	Y	Y	Y	Y	Y	Y	Y	Y	Y	Y	Y
	Are suggestions provided for further areas to research?	Y	Y	Y	Y	Y	Y	Y	Y	Y	Y	Y	Y
	Is there external validity?	Y	Y	N	Y	N	Y	N	Y	Y	Y	Y	Y

Appendix C: Details of CASP Critical Appraisal Checklist for Quantitative Studies Reviewed

CASP Critical Appraisal for Qualitative Studies Checklist	Buono et al.
Was there a clear statement of the aims of the research?	Y
Is a qualitative methodology appropriate?	Y
Was the research design appropriate to address the aims of the research?	Y
Was the recruitment strategy appropriate to the aims of the research?	Y
Was the data collected in a way that addressed the research issue?	Y
Has the relationship between researcher and participants been adequately considered?	N/A
Have ethical issues been taken into consideration?	Y
Was the data analysis sufficiently rigorous?	Y
Is there a clear statement of findings?	Y
How valuable is the research?	The research is valuable as it outlines the different procedures to achieve commissural alignment for each THV on a case-by-case basis, however, replication may be difficult as aortic anatomy and clinical situations differ in each patient.
